# Ezetimibe Induces Vasodilation in Rat Mesenteric Resistance Arteries through Inhibition of Extracellular Ca^2+^ Influx

**DOI:** 10.3390/ijms241813992

**Published:** 2023-09-12

**Authors:** Eun Yi Oh, Chae Eun Haam, Sooyeon Choi, Seonhee Byeon, Soo-Kyoung Choi, Young-Ho Lee

**Affiliations:** Department of Physiology, Yonsei University College of Medicine, 50 Yonseiro, Seodaemun-gu, Seoul 03722, Republic of Korea; ey5242@yonsei.ac.kr (E.Y.O.); cehaam@yonsei.ac.kr (C.E.H.); doh0902@yonsei.ac.kr (S.C.); seonhee89@yuhs.ac (S.B.)

**Keywords:** ezetimibe, mesenteric arteries, vasodilation

## Abstract

Ezetimibe is a lipid-lowering agent that selectively inhibits cholesterol absorption by binding to the Niemann–Pick C1-like 1 (NPC1L1) protein. Although it is well known that administration of ezetimibe in hypercholesterolemia patients reduces the risk of cardiovascular events through attenuation of atherosclerosis, studies on the direct effect of ezetimibe on vascular function are not sufficient. The aim of the present study was to investigate the vascular effects of ezetimibe in rat mesenteric arteries. In the present study, 12-week-old male Sprague Dawley rats were used. After the rats were sacrificed, the second branches of the mesenteric arteries were isolated and cut into 2–3 mm segments and mounted in a multi-wire myography system to measure isometric tension. Ezetimibe reduced vasoconstriction induced by U46619 (500 nM) in endothelium-intact and endothelium-denuded arteries. Ezetimibe-induced vasodilation was not affected by the endothelial nitric oxide synthase (eNOS) inhibitor N^ω^-Nitro-L-arginine (L-NNA, 300 μM) or the non-selective potassium channel blocker, tetraethylammonium (TEA, 10 mM). Moreover, ezetimibe also completely blocked the contraction induced by an increase in external calcium concentration. Ezetimibe significantly reduced vascular contraction induced by L-type Ca^2+^ channel activator (Bay K 8644, 30 nM). Treatment with ezetimibe decreased the phosphorylation level of 20 kDa myosin light chain (MLC_20_) in vascular smooth muscle cells. In the present study, we found that ezetimibe has a significant vasodilatory effect in rat mesenteric resistance arteries. These results suggest that ezetimibe may have beneficial cardiovascular effects beyond its cholesterol-lowering properties.

## 1. Introduction

Cardiovascular diseases (CVDs) refer to a broad spectrum of conditions linked to the heart and circulatory system, comprising coronary heart disease, cerebrovascular disease, atrial fibrillation, heart attacks, congenital heart issues, heart failure, and strokes [1]. The World Health Organization (WHO) classifies CVDs as the primary contributor to worldwide mortality and among the most urgent global health issues. In 2019, approximately 17.9 million individuals lost their lives to CVDs, accounting for 32% of all global fatalities [2].

Elevated plasma cholesterol is one of the major risk factors for CVDs [3]. The regulation of the plasma cholesterol level involves the contribution of endogenously synthesized cholesterol, dietary cholesterol intake, and the uptake of biliary cholesterol in the small intestine [4]. It is known that cholesterol contributes to disrupting and modifying vascular structure and function as it accumulates within the vascular wall lining. This interference can result in endothelial dysfunction, leading to the development of lesions, plaques, occlusions, and emboli, while also impairing the recovery and effective management of ischemia reperfusion injuries [5,6].

Given that elevated cholesterol levels are considered as an important risk factor for the development of CVDs, significant focus has been placed on examining the effects and mechanisms of cholesterol-lowering treatments [7,8]. Clinical studies have provided evidence indicating that decreasing plasma cholesterol level through dietary or pharmacological interventions can result in a decrease in the occurrence of cardiovascular disease-related fatalities [9]. Statins, ezetimibe, and novel proprotein convertase subtilisin/kexin type 9 (PCSK9) inhibitors are well known to improve lipid profiles and have beneficial effects on cardiovascular mortality [10,11,12]. Statins act by competitively inhibiting 3-hydroxy-3-methyl-glutaryl-coenzyme A (HMG-CoA) reductase, the pivotal enzyme in cholesterol biosynthesis [13]. In addition to their lipid lowering action, further pleiotropic effects were reported on vascular endothelial function and anti-inflammatory action [14]. The PCSK9 inhibitors are prescribed for patients who still have elevated cholesterol levels despite receiving statin treatment [15]. In recent studies, PCSK9 inhibitors like evolocumab and alirocumab have demonstrated substantial reductions in cardiovascular events, including myocardial infarction, stroke, and coronary revascularization. However, they have not shown a significant advantage in terms of reducing cardiovascular mortality [16,17].

Ezetimibe inhibits Niemann–Pick C1-like 1 (NPC1L1), which is a cholesterol transporter found in the apical membrane of intestinal enterocytes, consequently reducing the absorption of cholesterol in the intestines [18]. Ezetimibe is prescribed for patients who have contraindications to statins or experience statin intolerance. It is also used as an adjunct to statin therapy in cases where there is an inadequate reduction in LDL cholesterol levels [11]. Recently, it has been reported that ezetimibe also shows pleiotropic effects beyond cholesterol lowering in humans and animals [19,20]. Nochioka et al. documented that ezetimibe enhances endothelial function and suppresses Rho-kinase activity in humans, indicating potential novel anti-atherogenic properties [21]. Additionally, the favorable effect of ezetimibe on atherosclerosis development was observed in apolipoprotein E-deficient mice subjected to a high-fat diet [9]. Similarly, ezetimibe reduced the formation of atherosclerotic lesions in a diet-induced atherosclerosis model involving hypercholesterolemic rabbits [18]. Nevertheless, when compared to statins the pleiotropic effects of ezetimibe are relatively modest, and their clinical significance remains uncertain. Furthermore, although there are several studies on the cardiovascular benefits of ezetimibe, the effects of ezetimibe on the vascular function in small resistance arteries have not been studied well. Thus, in the present study, we evaluated the direct vascular effects of ezetimibe in rat mesenteric resistance arteries

## 2. Results

### 2.1. Effect of Ezetimibe on the Contraction Induced by Thromboxane Analogue, U46619

**Figure 1:**
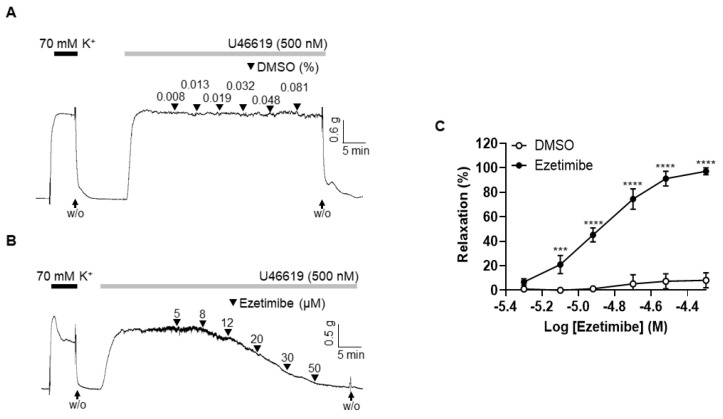
Ezetimibe-induced vasodilation in rat mesenteric arteries. (**A**) Data showing responses to vehicle (DMSO, 0.008–0.081%). (**B**) Representative trace demonstrating responses to cumulative administration of ezetimibe (5–50 µM) on U46619 (500 nM)-induced vasocontraction. (**C**) Statistical analysis of the relaxation response to U46619. Data are shown as mean ± SD (n = 5). W/O: wash out. *** *p* < 0.001, **** *p* < 0.0001.

Ezetimibe was administered to the mesenteric arteries pre-constricted with thromboxane analogue U46619 (500 nM) to explore its direct vascular effect. The vehicle, dimethyl sulfoxide (DMSO, 0.008–0.081%), did not change the U46619-induced vascular contraction (Figure 1A). Administration of ezetimibe (5–50 μM) concentration-dependently induced relaxation in the mesenteric arteries (Figure 1B). The maximal value of ezetimibe-induced vasodilation was 97.25 ± 2.82% in the arteries pre-contracted with U46619. The EC_50_ of ezetimibe was 16.5 μM (Figure 1C).

### 2.2. Endothelium-Independent Vasodilation Induced by Ezetimibe

**Figure 2:**
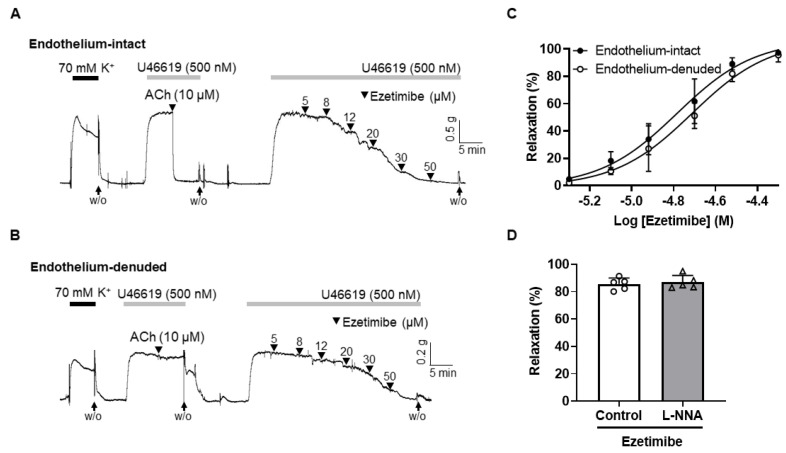
Endothelium-independent relaxation induced by ezetimibe in mesenteric arteries. Typical trace showing ezetimibe-induced relaxation in the endothelium-intact (**A**) and endothelium-denuded (**B**) mesenteric arteries. Statistical analysis of ezetimibe-induced vascular relaxation (**C**), and the vascular relaxation in response to ezetimibe in the presence of eNOS inhibitor L-NNA (300 µM, (**D**)). Mean ± SD (n = 5). ACh: acetylcholine; L-NNA: N^ω^-Nitro-L-arginine; W/O: wash out.

To investigate whether ezetimibe-induced vascular relaxation is dependent on endothelium, ezetimibe was treated in endothelium-intact (Figure 2A) or endothelium-denuded (Figure 2B) mesenteric arteries. Endothelial denudation was confirmed by the absence of relaxation when acetylcholine (10 μM) was applied to the arteries contracted with U46619 (500 nM). The maximal value of ezetimibe-induced vascular relaxation was 97.27 ± 1.72% in the endothelium intact arteries and 93.34 ± 6.31% in the endothelium denuded arteries. We did not observe a significant difference between the endothelium-intact and endothelium-denuded arteries (Figure 2C). To confirm the endothelium-independent vasodilatory effect of ezetimibe, and to further investigate whether the nitric oxide (NO) pathway is associated with ezetimibe-induced vasodilation, mesenteric arteries were incubated with the endothelial nitric oxide (eNOS) inhibitor N^ω^-Nitro-L-arginine (L-NNA, 300 µM) for 20 min before being contracted with U46619 (500 nM). The vasodilatory responses induced by ezetimibe were 85.57 ± 4.41% and 86.85 ± 4.97% in the presence and absence of L-NNA, respectively (Figure 2D).

### 2.3. Effect of Non-Specific K^+^ Channel Blocker, TEA, on the Vasodilation Induced by Ezetimibe

**Figure 3:**
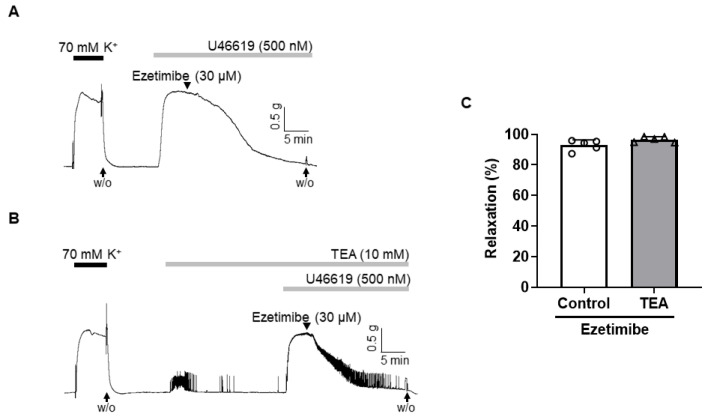
Effect of K^+^ channel blocker TEA (tetraethylammonium) on ezetimibe-induced vascular relaxation. Effects of ezetimibe on the pre-contracted arteries with U46619 (500 nM) in the presence (**A**) and absence (**B**) of TEA. (**C**) Statistical analysis of the relaxation response of ezetimibe in the presence of TEA. Vasodilation of arteries is expressed as the percentage of the contraction induced by U46619 (500 nM). Mean ± SD (n = 5). W/O: wash out.

To explore the potential role of K^+^ channels in ezetimibe-induced vasodilation, we pre-treated the arteries with a non-specific K^+^ channel blocker, tetraethylammonium (TEA, 10 mM), prior to inducing contraction with U46619 (500 nM). Notably, the administration of TEA did not affect the vasodilatory effect of ezetimibe in mesenteric arteries (Figure 3B). The vascular relaxation response induced by ezetimibe in the presence of TEA was 96.64 ± 1.82% (Figure 3C).

### 2.4. Effect of Ezetimibe on the Extracellular Ca^2+^-Induced Vascular Contraction

**Figure 4:**
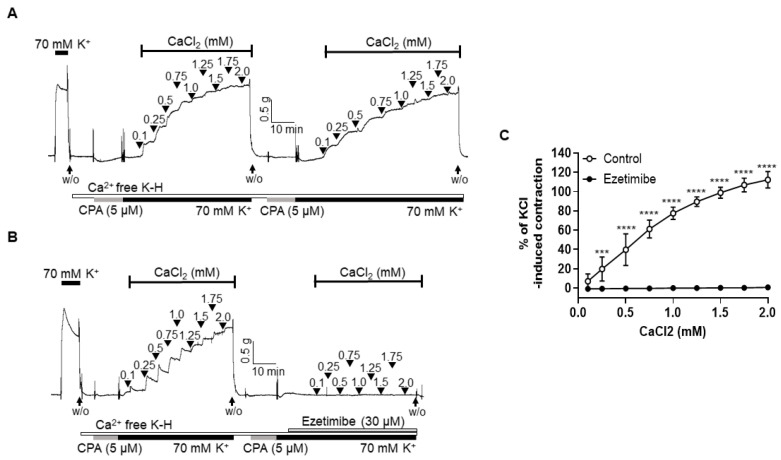
Ezetimibe inhibited vasocontraction induced by extracellular Ca^2+^. Representative traces showing the contraction responses by repeated addition of CaCl_2_ (0.1–2.0 mM) in the absence of ezetimibe (**A**) and in the presence (**B**) of ezetimibe (30 µM). (**C**) Statistical analysis of contraction induced by CaCl_2_ in the mesenteric arteries with or without ezetimibe. Mean ± SD (n = 5). W/O: wash out; CPA: cyclopiazonic acid. *** *p* < 0.001, **** *p* < 0.0001.

Since the suppression of K^+^ channels did not alter the response to ezetimibe, we proceeded to investigate whether ezetimibe’s relaxation effect is linked to the direct inhibition of extracellular Ca^2+^ influx. We induced contraction responses in the arteries by adding CaCl_2_ (ranging from 0.1 to 2.0 mM) while incubating them in a Ca^2+^-free K-H solution that contained the sarcoplasmic reticulum Ca^2+^-ATPase (SERCA) inhibitor cyclopiazonic acid (CPA, 5 µM) and KCl (70 mM). It was confirmed that the contraction responses caused by the repeated addition of CaCl_2_ were not changed (Figure 4A). Pre-treatment of ezetimibe (30 μM) significantly reduced the contractile responses induced by the cumulative addition of CaCl_2_ (Figure 4B,C).

### 2.5. Effect of Ezetimibe on the BAY K 8644-Induced Contraction

**Figure 5:**
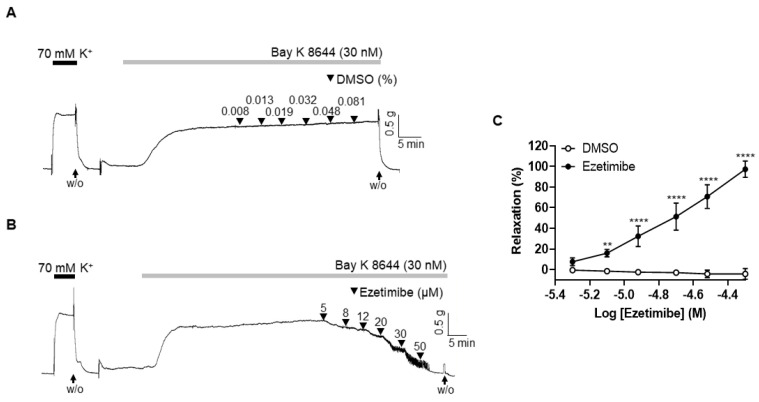
Ezetimibe reduced contraction induced by L-type voltage-gated calcium channel activation. Representative traces showing the effects of the vehicle (DMSO, (**A**)) and ezetimibe (**B**) on BAY K 8644-induced contraction. (**C**) Statistical analysis of relaxation induced by ezetimibe in the mesenteric arteries pre-constricted by BAY K 8644. Mean ± SD (n = 5). W/O: wash out. ** *p* < 0.01, **** *p* < 0.0001.

To investigate the correlation between ezetimibe-induced vasodilation and the reduction in extracellular Ca^2+^, arterial segments were exposed to K-H solution containing 15 mM of K^+^. This experimental environment facilitated the activation of voltage-gated calcium channels (VGCCs), subsequently followed by contraction induced by BAY K 8644 (30 nM), an L-type VGCC activator. Administration of the vehicle (DMSO, 0.008–0.081%) did not affect BAY K 8644-induced contraction (Figure 5A). Treatment of ezetimibe significantly reduced the vascular contraction induced by BAY K 8644 in a concentration-dependent manner (Figure 5B,C).

### 2.6. Effect of Ezetimibe on the Phosphorylation Level of MLC_20_ in VSMCs

**Figure 6:**
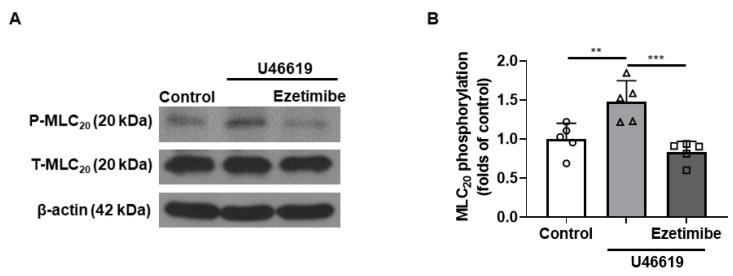
Ezetimibe decreased the phosphorylation of MLC_20_. (**A**) Representative western blot analysis of phosphorylated MLC_20_ (P-MLC_20_) and total MLC_20_ (T-MLC_20_) in control VSMCs, VSMCs treated with U46619 (500 nM), and VSMCs co-treated with U46619 (500 nM) and ezetimibe (30 µM). (**B**) Quantitative data for phosphorylated MLC_20_ (n = 5). ** *p* < 0.01, *** *p* < 0.001.

To investigate whether ezetimibe-induced vasodilation was caused by decreased phosphorylation of 20 kDa myosin light chain (MLC_20_), the phosphorylation and expression levels of MLC_20_ were observed in vascular smooth muscle cells (VSMCs, Figure 6). The administration of U46619 (500 nM) increased the phosphorylation level of MLC_20_ in VSMCs, which was decreased by the treatment with ezetimibe (30 μM).

## 3. Discussion

Hypercholesterolemia, defined as excessively high plasma cholesterol levels, is considered as a significant risk factor for developing a wide spectrum of CVDs, including coronary artery diseases, peripheral artery diseases, and stroke [22,23]. In the context of microcirculation, there is compelling evidence that the progression of hypercholesterolemia is closely linked to endothelial cell dysfunction [24,25]. Furthermore, studies have reported that hypercholesterolemia is associated with a significant reduction in vascular nitric oxide (NO) availability, increased oxidative stress, and the development of a highly inflammatory environment that can lead to severe disruptions in vascular reactivity [26,27]. Therefore, improving vascular endothelial function through cholesterol management contributes to better outcomes for individuals with CVDs.

Ezetimibe is a new selective cholesterol absorption inhibitor that inhibits the intestinal uptake and absorption of cholesterol [28]. In clinical practice, ezetimibe is often used in combination with statins to treat CVD patients [29]. It is known that treatment with ezetimibe in combination with statins improves coronary endothelial function in target vessels in coronary artery disease patients after coronary stenting [30]. However, no studies have directly examined the effects of ezetimibe alone on vascular function. Thus, the aim of this study was to explore the vascular effect of ezetimibe in rat mesenteric resistance arteries and to determine the underlying mechanism.

In the current study, we observed the vascular effect of ezetimibe in rat mesenteric resistance arteries. We found that ezetimibe induced vasodilation in a concentration-dependent manner in arteries pre-contracted with the thromboxane analogue U46619. Dilation of arteries occurs when the smooth muscle cells in the walls of arteries relax [31]. This relaxation can result from the elimination of a contractile stimulus or the direct influence of substances that induce vasodilation. The vascular endothelium, comprising a monolayer of endothelial cells that line the inner surface of blood vessels, plays a pivotal role in this process [32]. When exposed to various stimuli, the endothelium releases vasoconstrictive substances such as thromboxane A_2_ (TXA_2_) and endothelin-1 (ET-1) as well as vasodilatory substances, including prostacyclin (PGI_2_), nitric oxide (NO), and endothelium-derived hyperpolarizing factor (EDHF) [33]. In the present study, we investigated whether ezetimibe-induced vasodilation is dependent on endothelium (Figure 2). We observed that the removal of the endothelium did not affect the relaxation effect of ezetimibe in rat mesenteric arteries. To confirm that ezetimibe-induced vascular relaxation is endothelium-independent, we inhibited the production of NO, which is the most potent vasodilatory substance [34], with the eNOS inhibitor L-NNA (Figure 3A). Ezetimibe-induced vasodilation was not affected by the treatment with L-NNA. From these results, we assume that the ezetimibe-induced vasodilation occurs through an endothelium-independent relaxation mechanism. These findings are not in accordance with the previous study by Maekawa et al. which reported that treatment of ezetimibe improved endothelium-dependent NO-mediated relaxation in a rabbit jugular vein graft [35]. They showed that ezetimibe enhanced the function of endothelial NO through an increase in the endothelial Ca^2+^ level. Since they administered ezetimibe in vivo and then performed the experiment in the isolated blood vessels, it is plausible that the effects of ezetimibe on the blood vessels may not be direct, but rather mediated through other mechanisms. Furthermore, the animal species and blood vessels used in the experiment differed from those used in the present study. Therefore, we postulate that our findings might exhibit differences due to these reasons.

After we confirmed that ezetimibe-induced vascular relaxation is endothelium-independent, we explored whether the K^+^ channel is involved in the ezetimibe-induced vasodilation. The K^+^ channels play a pivotal role in determining and regulating the membrane potential of vascular smooth muscle cells [36]. This regulation of membrane potential by K^+^ channels affects the probability of VGCCs being in the open state, which in turn triggers the influx of Ca^2+^ and subsequent smooth muscle contraction. Conversely, membrane hyperpolarization facilitates the closure of VGCCs, impeding the entry of extracellular Ca^2+^ and promoting relaxation of the smooth muscle cells [37]. In the present study, we used the non-specific K^+^ channel blocker TEA to examine the involvement of the K^+^ channel in ezetimibe-induced vasodilation (Figure 3). Treatment with TEA did not alter the vasodilatory effect of ezetimibe, which indicates that the vasodilatory effect of ezetimibe is not related to the activation of the K^+^ channels.

After we found that the endothelium and K^+^ channels in smooth muscle cells are not involved in ezetimibe-induced vasodilation, we investigated whether ezetimibe directly affects the cytosolic Ca^2+^ level in smooth muscle cells. Since blockage of the Ca^2+^ channels could not induce enough contraction to test the effect of ezetimibe, instead of using a Ca^2+^ channel blocker, we incubated mesenteric arteries in a Ca^2+^-free K-H solution containing CPA to remove intracellular free Ca^2+^, then 70 mM K^+^ was administered to enable the opening of the VGCCs. The Ca^2+^-induced vasoconstriction was significantly reduced by pre-treatment with ezetimibe. In order to confirm that ezetimibe directly acts on the contraction induced by VGCC activation, we used the VGCC activator BAY K 8644. We found that ezetimibe induced vasodilation in mesenteric resistance arteries pre-contracted with BAY K 8644 (Figure 5). From these results we postulated that ezetimibe might inhibit extracellular Ca^2+^ influx in rat mesenteric arteries. Contraction of vascular smooth muscle cells is dependent on the level of 20 kDa myosin light chain (MLC_20_) phosphorylation. The MLC_20_ is phosphorylated by myosin light-chain kinase (MLCK), which is activated by an increase in intracellular Ca^2+^ [38]. Since we observed that ezetimibe reduced Ca^2+^-induced vascular contraction, we explored whether the phosphorylation level of MLC_20_ was affected by ezetimibe. We observed that administration of U46619 induced an increase in MLC_20_ phosphorylation, which was significantly reduced by treatment with ezetimibe (Figure 6). While we did not directly assess the alterations in the intracellular Ca^2+^ concentration induced by ezetimibe, our findings strongly imply that ezetimibe reduced phosphorylation of MLC_20_, which finally reflects the degree of contraction, resulting in vascular relaxation in rat mesenteric resistance arteries.

**Figure 7:**
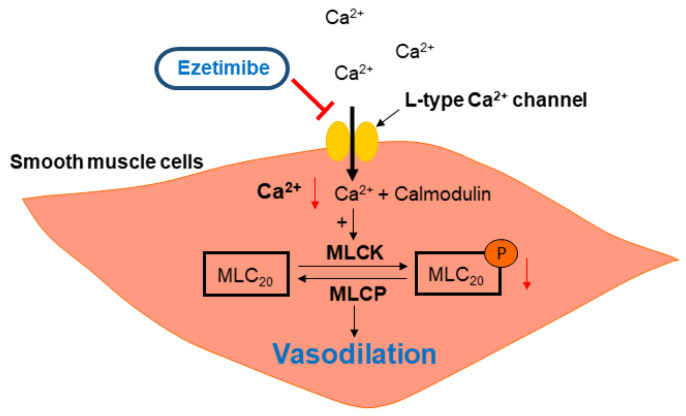
Graphical illustration of ezetimibe-induced vasodilation. MLC_20_: 20 kDa myosin light chain; MLCK: myosin light-chain kinase; MLCP: myosin light-chain phosphatase.

In the present study, for the first time, we reported the direct effect of ezetimibe on isolated mesenteric resistance arteries (Figure 7). Most of the studies published so far have observed the cardiovascular effects of ezetimibe through in vivo treatment. The direct effect of ezetimibe on resistance arteries has not been studied well. In this study, we demonstrated that ezetimibe induced vasodilation in mesenteric arteries contracted with an agonist, U46619. Furthermore, removal of endothelium and treatment with an eNOS inhibitor (L-NNA) did not affect the relaxation effect of ezetimibe. The non-specific K^+^ channel blocker TEA did not affect the ezetimibe-induced vasodilation either. Ezetimibe significantly inhibited BAY K 8644-induced vascular contraction, which was confirmed by Ca^2+^-induced contraction being inhibited by ezetimibe. Our discoveries contrast with previous studies that prioritize cholesterol reduction as the primary avenue for improving endothelial cell function. In addition to its cholesterol-lowering properties, ezetimibe exerts a direct effect on vascular smooth muscle cells, promoting blood vessel relaxation and enhancing overall vascular function improvement. This suggests potential mechanisms may exist related to the beneficial effect of ezetimibe. This novel finding can contribute to understanding the mechanism of action of ezetimibe, which is essential for treating CVD patients.

## 4. Materials and Methods

### 4.1. Animals

Ten to twelve-week-old male Sprague Dawley rats were housed in individually ventilated caging system cages and in controlled conditions with a light–dark cycle of 12:12 h, 50 ± 10% humidity, and 22 ± 2 °C. A total of 65 rats were used in this study. The animals had food pellets and water ad libitum.

### 4.2. Tissue Preparation

After the rats were sacrificed, the mesenteric resistance arteries were removed and placed in an ice-cold Krebs–Henseleit (K-H) solution (composition (mM): NaCl 119, NaHCO_3_ 25, glucose 11.1, KCl 4.6, MgSO_4_ 1.2, KH_2_PO_4_ 1.2, and CaCl_2_ 2.5) aerated with 95% O_2_ and 5% CO_2_. The fat tissues surrounding the mesenteric arteries were removed using forceps under a microscope (model SZ-40, Olympus, Shinjuku-ku, Tokyo, Japan). The second branches of the mesenteric arteries (diameter of 250–300 μm) were cut into 2–3 mm long segments.

### 4.3. Measurement of Isometric Tension in Mesenteric Arteries

The mesenteric artery segments were mounted in a wire myograph (model 620M, Danish Myotechnology, Aarhus, Denmark) to assess isometric tension. Mesenteric arterial rings were incubated in 37 °C K-H solution, aerated with 95% O_2_ and 5% CO_2_ for 30 min for equilibration, then stretched to their optimal resting tension (4 mN).

### 4.4. Experimental Protocols

The responses of the arteries were measured by contracting them using KCl (70 mM) or U46619 (500 nM), followed by the cumulative addition of ezetimibe (5–50 μM). The degree of relaxation response to ezetimibe was expressed as percent relaxation after preconstriction with U46619 (500 nM). To investigate whether the endothelium is involved in the vasodilatory response of ezetimibe, ezetimibe-induced relaxation was measured on endothelium-intact and endothelium-denuded mesenteric artery rings pre-contracted with U46619 (500 nM). Where indicated, the endothelium was removed by gently rubbing using forceps. The endothelium denudation was confirmed by the absence of relaxation in response to acetylcholine (ACh, 10 μM). To investigate the vascular mechanism of ezetimibe, L-NNA or TEA was pre-treated for 20 min, and then the relaxation response of ezetimibe was measured in the same arterial segments. To determine the involvement of Ca^2+^ influx in ezetimibe-induced relaxation, the normal K-H solution was replaced with Ca^2+^-free K-H solution containing 70 mM of KCl and CPA (5 μM), then CaCl_2_ was cumulatively added. Ezetimibe (30 μM) was treated in the same arteries 20 min before another cumulative addition of CaCl_2_. The CaCl_2_-induced contraction was calculated as percentage of maximum contraction induced by KCl (70 mM). To delineate the mechanism of ezetimibe-induced vasodilation, some arteries were pre-contracted using BAY K 8644 (30 nM) in K-H solution containing 15 mM KCl.

### 4.5. Isolation and Culture of Vascular Smooth Muscle Cells

After the rats were sacrificed, the aortas were excised, and the adipose and connective tissues were removed. The aortas were cut into small segments and transferred into a tube containing collagenase (1 mg/mL, Worthington Biomedical Corporation, Lakewood Town-ship, NJ, USA) and elastase (0.5 mg/mL, Calbiochem, San Diego, CA, USA) in Dulbecco’s Modified Eagle Medium (DMEM, Gibco, Waltham, MA, USA) at 37 °C for 30 min. After trituration and centrifugation, the cells were seeded in culture dishes (Corning, New York, NY, USA) and cultivated in DMEM supplemented with 10% fetal bovine serum (FBS), 100 IU/mL penicillin, and 10,000 µg/mL streptomycin (Gibco) at 37 °C in 5% CO_2_. The early passage cells (between two and four) were used in this study.

### 4.6. Western Blot Analysis

The cultured VSMCs were treated with a vehicle (0.05%, DMSO) or U46619 (500 nM) or U46619 (500 nM) with ezetimibe (30 µM) and, then, homogenized in an ice-cold lysis buffer, as described previously [39]. Western blot analysis was performed for the total MLC_20_ and phosphorylated MLC_20_ (1:1000 dilution; Cell Signaling, Boston, MA, USA). The blots were stripped and then re-probed with the β-actin antibody (1:3000 dilution; Santa Cruz Biotechnology, Santa Cruz, CA, USA) to verify the equal loading between the samples.

### 4.7. Chemicals and Reagents

U46619 was purchased from Cayman Chemical (Ann Arbor, MI, USA). Ezetimibe, acetylcholine (ACh), L-NNA, TEA, and general reagents were purchased from Sigma-Aldrich (St. Louis, MO, USA). CPA was obtained from Enzo Life Sciences (Farmingdale, NY, USA).

### 4.8. Statistical Analysis

All values are expressed as mean ± standard deviations. The n-values in Figure 1, Figure 2, Figure 3, Figure 4 and Figure 5 mean number of arteries derived from each different animal. The n-value in Figure 6 means number of experiments with pooled samples. Comparison among groups was analyzed by two-way repeated measure ANOVA followed by Bonferroni’s test in Figure 1, Figure 2C, Figure 4 and Figure 5. For Figure 2D, we used two-way ANOVA. For Figure 3, an unpaired Student’s t-test was used. For Figure 6, one-way ANOVA followed by Bonferroni’s test was used. Statistical analysis was performed using GraphPad Prism (Version 7, GraphPad software, La Jolla, CA, USA).

## Data Availability

Not applicable.
